# Lightweight semantic segmentation network for tumor cell nuclei and skin lesion

**DOI:** 10.3389/fonc.2024.1254705

**Published:** 2024-03-27

**Authors:** Yan Chen, Xiaoming Sun, Yan Duan, Yongliang Wang, Junkai Zhang, Yuemin Zhu

**Affiliations:** ^1^ Heilongjiang Province Key Laboratory of Laser Spectroscopy Technology and Application, Harbin University of Science and Technology, Harbin, China; ^2^ INSA Lyon, University Claude Bernard Lyon 1, CNRS, Inserm, CREATIS UMR 5220, U1294, Lyon, France

**Keywords:** semantic segmentation, tumor cell nuclei, skin lesions, attention mechanism, feature extraction

## Abstract

In the field of medical image segmentation, achieving fast and accurate semantic segmentation of tumor cell nuclei and skin lesions is of significant importance. However, the considerable variations in skin lesion forms and cell types pose challenges to attaining high network accuracy and robustness. Additionally, as network depth increases, the growing parameter size and computational complexity make practical implementation difficult. To address these issues, this paper proposes MD-UNet, a fast cell nucleus segmentation network that integrates Tokenized Multi-Layer Perceptron modules, attention mechanisms, and Inception structures. Firstly, tokenized MLP modules are employed to label and project convolutional features, reducing computational complexity. Secondly, the paper introduces Depthwise Attention blocks and Multi-layer Feature Extraction modules. The Depthwise Attention blocks eliminate irrelevant and noisy responses from coarse-scale extracted information, serving as alternatives to skip connections in the UNet architecture. The Multi-layer Feature Extraction modules capture a wider range of high-level and low-level semantic features during decoding and facilitate feature fusion. The proposed MD-UNet approach is evaluated on two datasets: the International Skin Imaging Collaboration (ISIC2018) dataset and the PanNuke dataset. The experimental results demonstrate that MD-UNet achieves the best performance on both datasets.

## 1 Introduction

The rapid segmentation of Tumor cell nuclei and skin lesion is a crucial technique in the field of medicine, contributing to more accurate disease diagnosis for doctors and patients. By processing and analyzing medical images, various information such as the location, size, shape, and density of lesions can be extracted, providing a basis for physicians to develop more scientifically informed treatment plans. Currently, research on semantic segmentation of medical images primarily focuses on two directions: traditional methods and deep learning-based methods. Traditional approaches rely on image processing and computer vision techniques such as edge detection, region growing, and threshold segmentation. These methods offer advantages such as fast computation speed and minimal data requirements. However, they are limited by the need for manual design and parameter adjustment, resulting in unstable performance across different images and tasks.

In recent years, deep learning-based segmentation methods have been at the forefront of research. Among them, UNet (1) is a representative deep learning network. UNet adopts an encoder-decoder architecture, where the encoder is responsible for extracting image features, and the decoder maps these features back to the original image size to generate segmentation masks. Additionally, UNet incorporates skip connections, which combine features from the encoder with those from the decoder, ensuring accurate and robust segmentation even with small datasets. Building upon UNet, several excellent network structures have been developed. U-Net++ (2) introduces nested and dense skip connections from DenseNet, further strengthening the skip connections and reducing the semantic gap between the encoder and decoder. U-Net 3+ (3) includes feature maps from both smaller and equivalent scales of the encoder, as well as feature maps from larger scales of the decoder, capturing fine-grained details and coarse-grained semantics across the entire feature map. Gudhe et al. (4) design the Multi-Level Dilated Residual (MLDR) blocks to replace the convolutional blocks in the classic U-Net, enhancing the learning capability. Xiao et al. (5) propose the Weighted Residual U-Net network, which replaces each layer of the encoder with residual connections to avoid or minimize the loss of natural information during image contraction. It also introduces a weighted attention mechanism that focuses only on the target region of interest and discards irrelevant noisy backgrounds. Luo et al. (6) introduce the weighted attention mechanism into the U-Net network and incorporate a Dense Connection Network (7), proposing the AD-UNet network to improve the utilization of model feature information while reducing network complexity and learning parameter complexity. Liu et al. (8) build upon the U-Net network with ResNet50 convolutional blocks and use a feature pyramid network to obtain segmentation outputs at different scales from the decoder. Jethi et al. (9) draw inspiration from domain transformation and propose a novel U-Net network structure with dual encoders and a single decoder for MRI image analysis. Dong et al. (10) propose an 8-layer U-Net automatic segmentation network based on a 4-layer U-Net network, aiming to extract deeper semantic features. He et al. (11) address the issue of non-smooth neighborhoods in pixel-level prediction caused by low tissue contrast in CT images. They propose the MetricUNet network based on metric learning, considering the relationships among voxel-level features in the images to achieve more precise segmentation results.

In addition, methods such as SegNet (12), UCTransNet (13), and R2UNet (14) have been proposed, achieving promising results in medical image segmentation. However, their research primarily focuses on enhancing network performance. In clinical practice, the rapid and accurate processing of medical images is crucial. To alleviate the healthcare burden brought about by population growth, some devices have transitioned from the laboratory to the point of care. This means that patients no longer need to queue for laboratory examinations as medical equipment can be brought directly to them (15). Point-of-care imaging aids clinicians in expanding their service options and improving patient care, reducing the time and steps involved in patients visiting radiology centers. Some devices can even detect bodily conditions using smartphones. Technological advancements centered around point-of-care imaging are enhancing patient satisfaction. In recent years, the application of point-of-care devices has steadily increased. For example, individuals can capture photos of their skin, hair, or nails from different angles using their smartphone camera and then utilize AI-assisted tools to analyze clinical images and relevant medical histories (16) in order to understand their own skin conditions. When individuals are bitten by mosquitoes outdoors, the motion of fluorescent nanoparticles in the blood, known as Brownian motion, can be measured using particle diffusometry (PD) (17). By combining PD with loop-mediated isothermal amplification (LAMP) technology on a smartphone, it becomes possible to determine whether the individual is infected with malaria. The process can be conveniently executed by capturing a 30-second video of the blood using a smartphone. Point-of-care ultrasound (POCUS) devices (18) enable physicians to perform ultrasound examinations at the patient’s bedside and conduct real-time analysis and diagnosis using smartphone applications. Remote guidance allows for real-time image recognition through text messages or email. Nalan Kozaci et al. conducted experiments comparing the accuracy of point-of-care ultrasound and X-ray examinations in diagnosing knee joint fractures. The experimental results demonstrated the effectiveness of POCUS examinations in detecting knee joint bone injuries (19). Swoop, the world’s first deep learning-based MR imaging system, provides neurological imaging at the point of care (20). This system can complete scans in under three minutes, enabling healthcare decision-making without transferring patients to radiology departments. These latest advancements in diagnostic technologies facilitate the rapid acquisition of clear images at the point of care. These devices also integrate tasks such as segmentation, classification, and registration to expedite the diagnostic process for both patients and clinicians. Integrating CNN and vision transformer can potentially enhance model performance by effectively capturing both local and global features (21). Dhamija et al. (22) propose two deep learning-based models, USegTransformer-P and USegTransformer-S, which merge transformer-based encoders and convolution based encoders to adequately extract global and local features. However, existing solutions such as UNet, MedT (23) and Cenet (24) still suffer from parameter redundancy and significant computational loads, posing challenges for real-time point-of-care applications.

The motivation of this study is to try to propose a model that has the following three characteristics: i) Accuracy, ii) A small number of parameters, and iii) A lower computational complexity, which is not the case for existing state-of-the-art network models. This would make the proposed MD-UNet specifically suitable for efficient inference and deployment in resource-constrained environments, such as mobile applications, embedded systems, and real-time applications. However, the presence of diverse tumor cell nuclei types and varied skin lesions poses challenges to the network’s robustness and segmentation accuracy. To address the computational issue, this paper draws inspiration from MLP-based networks (25–29), specifically UNeXt (29), as the first MLP-based network capable of matching Transformer performance while requiring fewer computational resources. Nonetheless, this paper still adopts a 5-layer U-Net as the backbone encoder-decoder structure. The traditional convolutional structure is replaced by the Tokenized Multi-Layer Perceptron (Token-MLP) architecture, which maps convolutional features to abstract tokens. Subsequently, MLPs are utilized to learn these tokens for segmentation, enabling the learning of semantic information at different levels. To enhance the segmentation accuracy of the network, this paper introduces Multi-layer Feature Extraction (MFE) modules inspired by attention mechanisms (30) and the Inception structure (31), effectively extracting semantic features of objects with different shapes in the encoder part. Moreover, to prevent semantic information loss and gradient vanishing, a Depthwise Attention (DA) block is designed to replace skip connections during the sampling process, efficiently integrating semantic information from both the encoder and decoder ends. **Table 1** summarizes the advantages and limitations of the above-mentioned networks.

**Table 1:** Advantages and limitations of the above-mentioned networks.

Networks	Advantages	Limitations
UNet (1)	- End-to-end fully convolutional network, no complex pre/post-processing needed - Can be trained with few training samples - Learning of simultaneous contextual and localization information	- Difficulty processing images with large target changes - Loss of spatial information
UNet++ (2)	Reduction of semantic gap between encoder and decoder owing to tested dense skip connections	- Large number of parameters and floating-point operations - Network structure is complex
U-Net3+ (3)	- Integration of multi-scale features via full-scale skip connections - Full-scale deep supervision for hierarchical representations - Need less parameters while being efficient	Full-scale skip connections result in an excessive redundancy in feature maps, leading to higher network memory consumption and computational load
Gudhe et al. (4)	- Multi-level dilated residual convolutions - Robust against outliers - Preserves better continuity in boundaries	Inaccurate edge segmentation
Xiao et al. (5)	- Using weighted attention mechanism, our model will only pay attention at the target area and discard the irrelevant noisy background.	- Complex image preprocessing - Lacks comparisons with existing approaches, and mentions of limitations or challenges
Luo et al. (6)	- Reduces the number of network parameters - Suppresses the overfitting of small datasets and mitigates the vanishing gradient phenomenon	Unsmooth edges appear in the segmentation results for the target areas.
Francia et al. (7)	- Fully double convolutional neural network - Shorter training time	- Complex image preprocessing - Complex network structure
Liu et al. (8)	- Feature pyramid network architecture is applied to extracting rich multi-scale features - High accuracy was achieved with a low number of epochs	Lacks comparisons with existing approaches, and mentions of limitations or challenges
Jethi et al. (9)	- Encoder single decoder-based architecture - By simultaneously optimizing both the raw kspace data and undersampled image data for reconstruction.	Complex network structure
Dong et al. (10)	- 8-layer network replaces the original 4-layer network to extract deeper image features - MeshGrid-Flip-Rotate augmentation improves network accuracy	- Large number of parameters - Large number of floating point operations
He et al. (11)	Modeling the inter-voxel relationships via voxel-level feature embeddings	Computational efficiency should be further optimized
Segnet (12)	Decoder uses pooling indices computed in the max-pooling step of the corresponding encoder to perform non-linear upsampling	- Large number of parameters - Large number of floating point operations
UCTransNet (13)	- Channel-wise Cross Fusion Transformer for encoder feature transformation - Channel-wise Cross Attention for feature fusion in decoder	- Large number of floating point operations - Difficulty in tuning hyperparameters
R2Unet (14)	- Residual unit helps when training deep architecture - Feature accumulation with recurrent residual convolutional layers ensures better feature representation	- Large number of parameters - Large number of floating point operations
Dhamija et al. (22)	Combine the Transformer model and CNN model to understand local features and global background	Complex network structure
MedT (23)	- Gated Axial attention - Small number of parameters - Local-Global training strategy	- Large number of floating point operations - Necessitates a large dataset - High computation time
Cenet (24)	- Dense Atrous Convolution and Residual Multi-kernel Pooling - Universal segmentation framework - Small number of floating point operations	Large number of parameters
Yu et al. (25)	- Simple network architecture - Less number of parameters - Less number of floating point operations	Easy to overfit
UNeXt (29)	- Faster inference - Reduced complexity - Less number of parameters - High segmentation accuracy	Easy to overfit
Atttention UNet (30)	- Attention Gate - High segmentation accuracy	Large number of floating point operations
Szegedy et al. (31)	- Scale residuals - Inception Module combines with Residual Connection to accelerate training	Complex network structure

The main research contributions of this paper can be summarized as follows:

- This paper employ Token-MLP as a replacement for traditional convolutional modules, aiming to capture dependency relationships within tokenized sequences by incorporating tokenization into the MLP architecture.
- This paper proposes a MFE module that combines channel attention, spatial attention and asymmetric convolution to enhance the effectiveness of feature extraction in the network and improve feature representation capabilities.
- This paper proposes the DA Block and integrates it into the skip connections of the U-shaped network to alleviate semantic ambiguity and enhance focus on lesions of interest.
- This paper trains, validates and tests our architecture on the ISIC2018 dataset (32) and the PanNuke dataset (33). Experiments show that the network we designed outperforms the baseline model and previous segmentation methods in terms of IoU, Dice, parameters and FLOPs, and provides a new research idea for medical image segmentation.

## 2 Method

**Figure 1:**
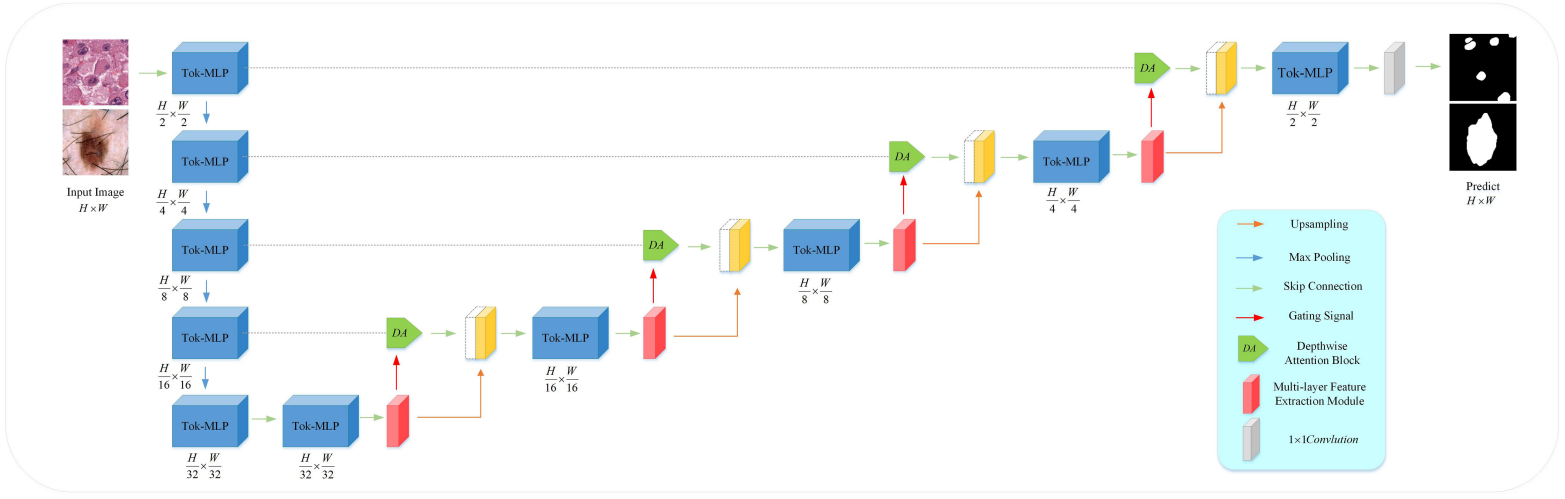
Overview of the proposed MD-UNet architecture.

MD-UNet adopts a 5-layer encoder-decoder architecture within the U-Net framework, as illustrated in **Figure 1**. In contrast to conventional convolutional operations, we propose the utilization of Token-MLP modules as a viable alternative. These Token-MLP modules offer several advantages, including parameter reduction, computational complexity reduction, and enhanced feature modeling capabilities (29). In the decoder, we employ a MFE module to extract features relevant to tumor cell nuclei and skin lesions. By integrating attention mechanisms and Inception structures, the network demonstrates improved segmentation accuracy and robustness. To better integrate semantic information from both the encoder and decoder ends, we introduce the DA block in the skip connections, which concurrently attends to semantic information from the encoding and decoding stages. By incorporating Depthwise Convolution (DWConv), we enhance the extraction and fusion of semantic features in both the encoding and decoding stages. The semantic information outputted by the DA block is combined with the semantic information outputted by the MFE model, providing semantic information for the subsequent layer. The subsequent section provides an overview of each module.

### 2.1 Token-MLP block

**Figure 2:**
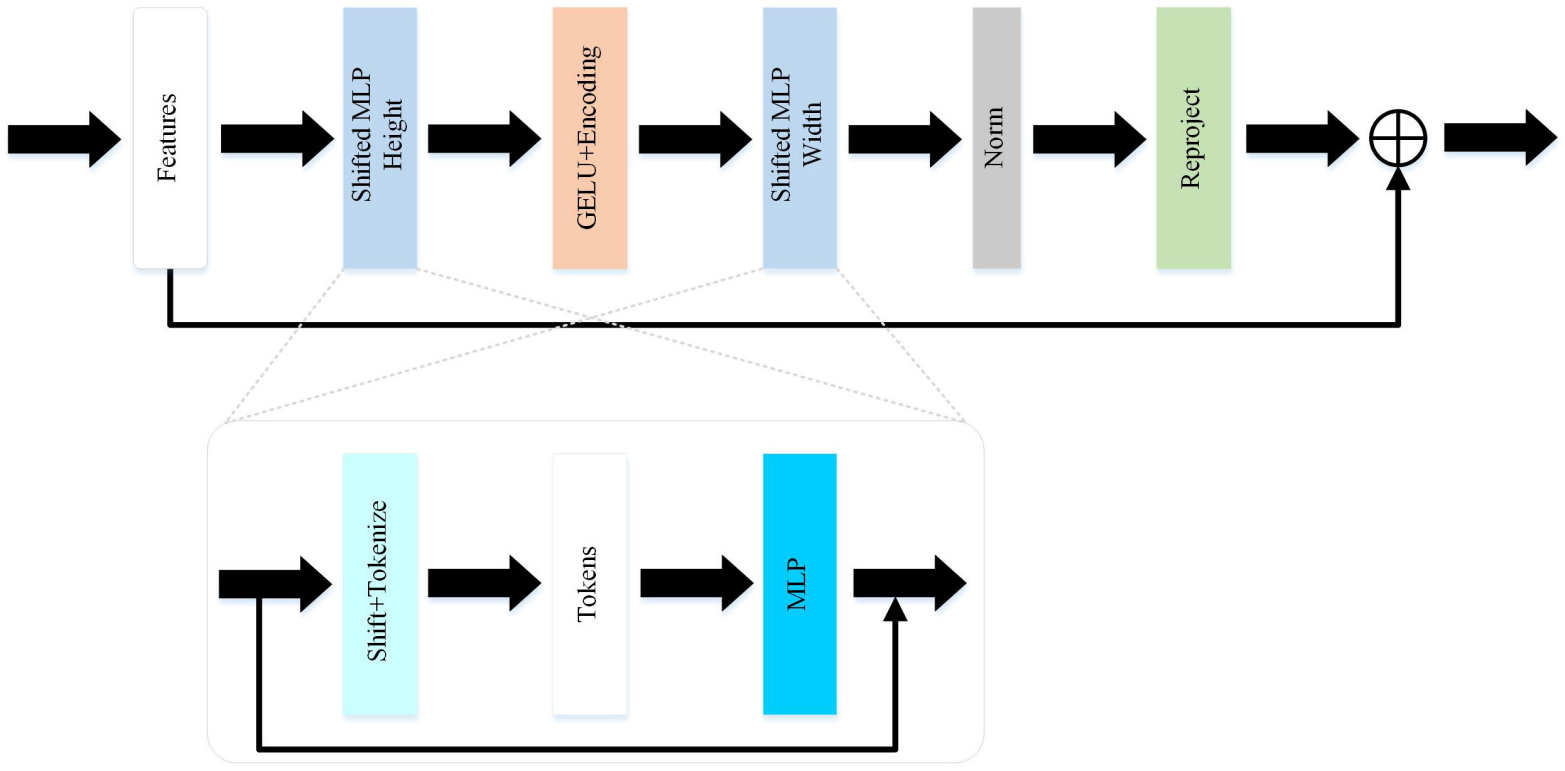
The token-MLP block in MD-UNet.

Compared to the UNet and improved versions of U-shaped networks, this paper selects the Token-MLP module to replace the conventional combination of convolution, batch normalization, and ReLU, as shown in **Figure 2**. By incorporating the advantages of the Swin transformer (34) and axial attention (35), this module integrates two shifted MLP modules to independently shift features along the height and width dimensions, partitioning them into distinct partitions and performing positional shifts along the specified axis. The objective of this design is to introduce local contextual information and enhance the module’s perceptual capability in feature processing.

The Token-MLP module operates by translating and projecting features, transforming them into a series of tokens. Initially, a convolutional kernel with a size of 3 is used to perform translation operations on the features, while adjusting the channel count to the embedding dimension E, where E represents the number of tokens. Subsequently, these tokens are processed through a shifted MLP module. By shifting features along both the height and width dimensions and dividing them into different partitions, the module effectively introduces local context and enhances its ability to perceive local features through the creation of random windows.

Furthermore, the Token-MLP module introduces improvements through the integration of residual connections. Within the shifted MLP module, residual connections are incorporated by adding the original token features as residuals to the final output. Such residual connections facilitate gradient propagation, addressing issues such as gradient vanishing and explosion. Moreover, they enable the network to better learn low-level features and enhance the expressive capacity of the network.

### 2.2 Multi-layer feature extraction module

**Figure 3:**
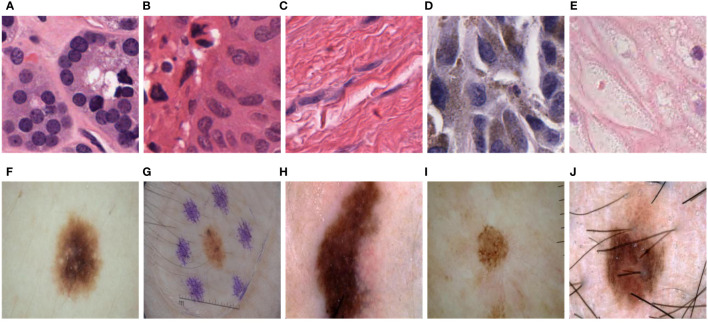
Variations in size, shape, color, and distribution among different tumor cell nuclei and skin lesions. **(A)**. Circular cell nuclei; **(B)**. Circular cell nuclei; **(C)**. Filamentous cell nuclei; **(D)**. Elliptical cell nuclei; **(E)**. Circular cell nuclei; **(F)**. Skin lesions display lighter colors; **(G)**. Skin lesions may be influenced by hair and artificial markings; **(H)**. Skin lesions exhibit darker shades; **(I)**. Skin lesions display lighter colors; **(J)**. Skin lesions may be influenced by hair and artificial markings.

The PanNuke dataset consists of H&E-stained images of 19 cell types, as shown in **Figure 3**. Due to the variations in cell types, the cell nuclei exhibit significant differences in shape, size, and color, especially in shape. Some cell nuclei are circular (**Figures 3A, B, E**), some are elliptical (**Figure 3D**), and there are even filamentous cell nuclei (**Figure 3C**). The ISIC2018 dataset also showcases the diversity of skin lesions. Some skin lesions have lighter colors (**Figure 3I**), while others have darker shades (**Figures 3H, F**). Additionally, certain lesions may be affected by hair and artificial markings (**Figures 3G, J**). Therefore, achieving high computational accuracy and robustness for the network is a major challenge.

To further enhance the accuracy of semantic segmentation, Drawing inspiration from spatial attention mechanisms (36) and cross-channel attention mechanisms (37), this study proposes a MFE module that extracts high-level and low-level semantic information in the network’s decoder. In this module, we integrate spatial attention mechanisms, cross-channel attention mechanisms, and asymmetric convolutions to enhance the network’s ability to extract semantic features, identify common characteristics among different types of cells or skin lesions, and improve the robustness of network segmentation.

**Figure 4:**
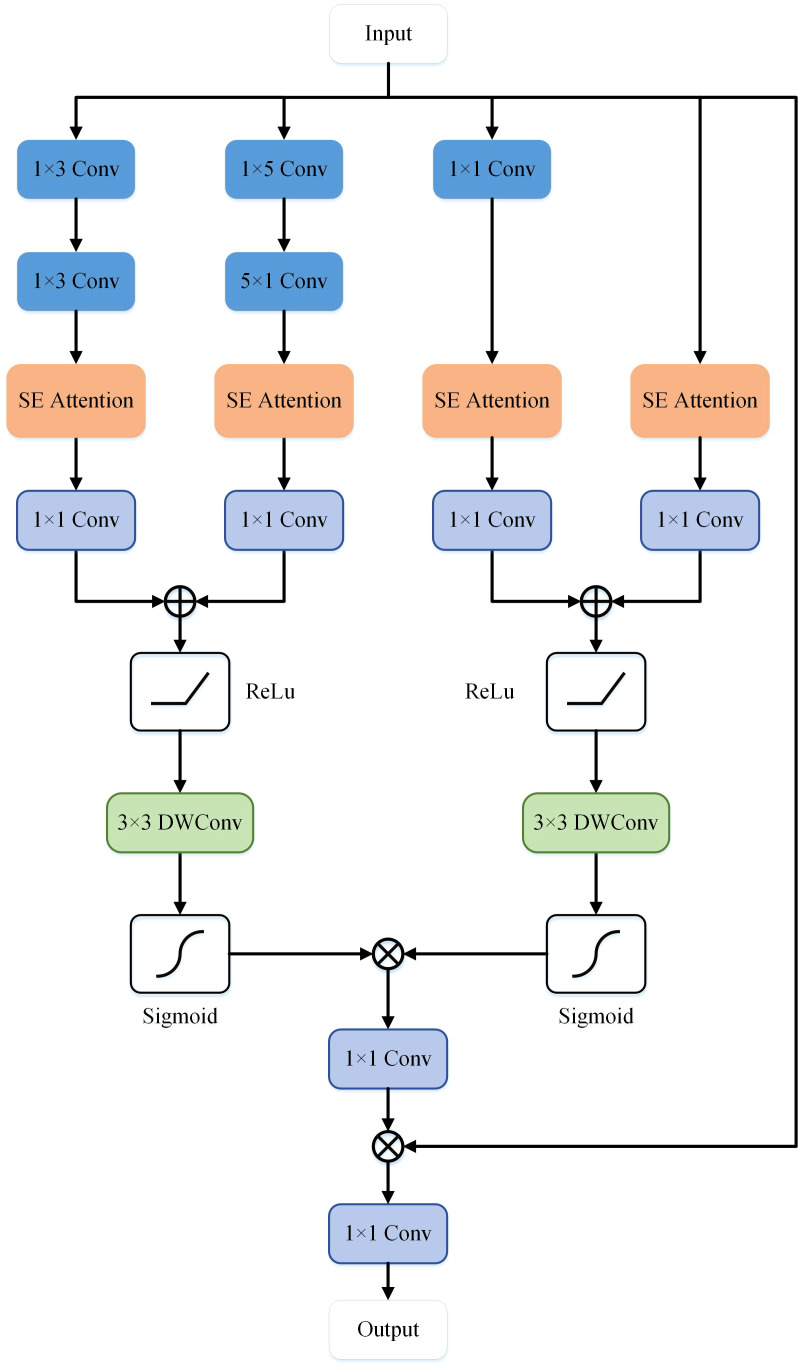
The structure of the Multi-layer Feature Extraction module in MD-UNet.


As illustrated in **Figure 4**, the MFE module framework comprises a total of five branches, with two branches utilizing skip connections and an additional two employing asymmetric convolutions. A combination of 1×1 convolution, 1×3 convolution, 3×1 convolution, 1×5 convolution, and 5×1 convolution is applied to the feature x to capture semantic information of objects with diverse shapes. The extracted feature information is fused and input into the Squeeze-and-Excitation (SE) module (37), establishing relationships and dependency models among channels. Subsequently, the feature map undergoes non-linear transformations through a 1x1 convolutional layer, generating four separate branches ${\text{C}}_{i}^{1}$ , ${\text{C}}_{i}^{2}$ , ${\text{C}}_{i}^{3}$ , ${\text{C}}_{i}^{4}$ , as depicted in Equation 1.

(1) $$\begin{array}{l} {\text{C}}_{i}^{1}={\text{W}}_{1,1}^{T}({\text{F}}_{SE}(\text{x}*{\text{W}}_{1,3}^{T}*{\text{W}}_{3,1}^{T}))\\ {\text{C}}_{i}^{2}={\text{W}}_{1,1}^{T}({\text{F}}_{SE}(\text{x}*{\text{W}}_{1,5}^{T}*{\text{W}}_{5,1}^{T}))\\ {\text{C}}_{i}^{3}={\text{W}}_{1,1}^{T}({\text{F}}_{SE}(\text{x}*{\text{W}}_{1,1}^{T}))\\ {\text{C}}_{i}^{4}={\text{W}}_{1,1}^{T}({\text{F}}_{SE}(\text{x})) \end{array}$$

Where x represents the semantic features output by the Token-MLP module on the decoding side. ${\text{W}}_{1,1}^{\text{T}}$ , ${\text{W}}_{1,3}^{\text{T}}$ , ${\text{W}}_{3,1}^{\text{T}}$ , ${\text{W}}_{1,5}^{\text{T}}$ , and ${\text{W}}_{5,1}^{\text{T}}$ respectively denote 1×1 convolution, 1×3 convolution,  3×1 convolution,  1×5 convolution, and 5×1 convolution. ${\text{F}}_{\text{SE}}\left(\text{x}\right)$ represents the Squeeze and Excitation operation. * denotes the convolution operation. The semantic information extracted from the four branches is subsequently connected and re-encoded through two spatial attention mechanisms, resulting in the generation of ${\text{O}}_{i}^{1}$ and ${\text{O}}_{i}^{2}$ , as illustrated in Equation 2.


(2) $$\begin{array}{l} {\text{O}}_{i}^{1}=\sigma_{2}({\text{W}}_{3,3}^{T}(\sigma_{1}({\text{C}}_{i}^{1}(\text{x})+{\text{C}}_{i}^{2}(\text{x}))))\\ {\text{O}}_{i}^{2}=\sigma_{2}({\text{W}}_{3,3}^{T}(\sigma_{1}({\text{C}}_{i}^{3}(\text{x})+{\text{C}}_{i}^{4}(\text{x})))) \end{array}$$

Where ${\text{σ}}_{1}\left(\text{x}\right)=\text{max}\left(0,\text{x}\right)$ corresponds to the ReLU activation function. ${\text{σ}}_{2}\left({\text{x}}_{\text{i},\text{c}}\right)=\frac{1}{1+\text{exp}\left(-{\text{x}}_{\text{i},\text{c}}\right)}$ corresponds to the sigmoid function. ${\text{ W}}_{3,3}^{\text{T}}$ respectively denote 3×3 depthwise convolution. Finally, non-linearity is enhanced through a 1×1 convolution, followed by element-wise multiplication with the input feature map, and then processed through another 1×1 convolution to obtain the ultimate output feature map, denoted as ${\hat{O}}_{\text{i}}$ , as illustrated in Equation 3.


(3) $${\hat{O}}_{\text{i}}={\text{W}}_{1,1}^{\text{T}}\left({\text{W}}_{1,1}^{\text{T}}\left({\text{O}}_{i}^{1}\cdot {\text{O}}_{i}^{2}\right)\cdot \text{x}\right)$$

### 2.3 Depthwise attention block

**Figure 5:**
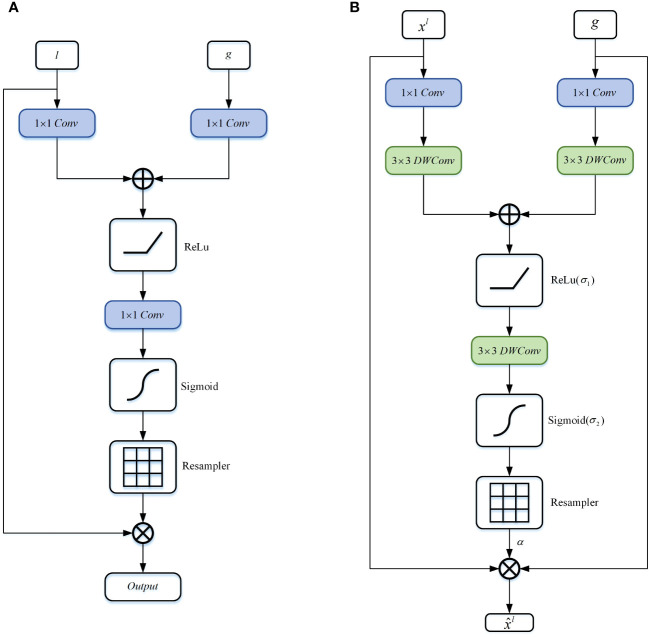
Schematic diagram of the DA block proposed in this article. **(A)** Attention Gate, **(B)** Depthwise Attention Block.

In order to accurately segment and predict target objects, the standard CNN architecture gradually downsamples the feature map grid to capture semantic contextual relationships. However, for small objects with significant shape variations, reducing false positive predictions solely through skip connections becomes challenging. Inspired by the Attention Gate (30), this paper proposes a DA block to replace the skip connection part of the U-shaped network. As shown in **Figure 5**, in contrast to the Attention Gate (**Figure 5A**), the DA block adopts a symmetrical structure (**Figure 5B**) that not only focuses on the coarse-grained semantic information from the encoder end but also pays attention to the fine-grained semantic information from the decoder end. Additionally, the introduction of DWConv enhances the accuracy and generalization ability of the model while encoding positional information of the encoded features. According to the study by (38), the convolutional layers within the MLP block sufficiently encode positional information and outperform standard positional encoding methods. Moreover, DWConv has fewer parameters and computational costs, making it relatively computationally efficient.

Specifically, firstly, the features $x^{l}$ and g undergo non-linear transformations through consecutive 1×1 convolutions and 3×3 DWConv. The transformed vectors of $x^{l}$ and g are then added together, and the result is passed through the ReLU activation function to retain significant activations. Subsequently, the feature map is propagated through a 3×3 depthwise convolutional layer to generate $C_{i}$ , as shown in Equation 4.


(4) $$C_{i}=W_{3}^{T}\left(\sigma_{1}\left(x^{l}*W_{x,1}^{T}*W_{x,3}^{T}+g*W_{g,1}^{T}*W_{g,3}^{T}+b_{i}\right)\right)$$

Where $x^{l}$ represents the coarse-grained semantic information gradually extracted through layer-wise processing of local information by the Token-MLP module, while g represents the fine-grained semantic information extracted at the decoder end by the MFE module. $W_{x,1}^{T}$ and $W_{g,1}^{T}$ denote 1×1 convolutions, and $W_{x,3}^{T}$ , $W_{g,3}^{T}$ and $W_{3}^{T}$ represent 3×3 depthwise convolutions. The term $b_{i}$ represents the bias term. Additive attention (39) is employed to obtain gating coefficients, which has been experimentally shown to achieve higher accuracy compared to multiplicative attention (40), despite being computationally more expensive.

Subsequently, we apply the sigmoid function to transform $C_{i}$ into the non-linear space, as shown in Equation 5.


(5) $$\alpha_{i}^{l}=\sigma_{2}\left(C_{i}\left(x_{i}^{l},{\text{g}}_{i};{\text{Θ}}_{att}\right)\right)$$

The DA block is characterized by a set of parameters ${\text{Θ}}_{att}$ , including $W_{x}\in {\mathbb{R}}^{F_{l}\times F_{int}}$ , $W_{g}\in {\mathbb{R}}^{F_{g}\times F_{int}}$ , $\text{ψ}\in {\mathbb{R}}^{F_{int}\times 1}$ , bias terms $b_{\text{ψ}}\in \mathbb{R}$ , $b_{\text{g}}\in {\mathbb{R}}^{F_{int}}$ . The features $x^{l}$ and g are nonlinearly mapped to the R-dimensional intermediate space, which is called attention based on vector cascading (41).

Finally, we compute the element-wise multiplication between the input feature map and the attention coefficients to obtain the final output of the DA block, as shown in Equation 6:


(6) $${\hat{x}}_{i,c}^{l}=x_{i,c}^{l}\cdot \alpha_{i}^{l}\cdot {\text{g}}_{i,c}$$

Where i and c represent the spatial and channel dimensions.

## 3 Experiments and results

### 3.1 Dataset

This paper used the International Skin Imaging Collaboration (ISIC2018) (32) and PanNuke (33). The ISIC2018 dataset is a large medical image dataset for skin lesion detection and classification tasks. It contains 2693 skin lesion images from various locations around the world, including malignant melanoma, benign melanocytic nevi, and other benign skin lesions. Each image is manually annotated by professional dermatologists and provides corresponding pathological diagnosis and classification information. PanNuke is an open-source Pan-Cancer histology dataset used for classifying and segmenting nuclei instances. The dataset presents a semi-automatically generated collection of exhaustive nuclei labels over 19 diverse tissue types. The dataset comprises 7825 images. Since the initial input images do not have the same size, before feeding the data directly into the network, we reformatted them into the same size. For the PanNuke dataset, we employed a cropping technique, which aims to fully retain essential information of the images, to crop all the initial large-size images into images of the same size 256×256. For the ISIC2018 dataset, we utilized an image resizing method to adjust all the images into the same 256×256 size.

We divided the dataset into training and testing sets in a 9:1 ratio. Moreover, within each training epoch, we utilized a random allocation technique at an 8:1 ratio to distribute data between the training and validation sets. More precisely, in the case of the PanNuke dataset, the training set encompassed 7043 samples, while the testing set comprised 728 samples. In the case of the ISIC2018 dataset, the training set consisted of 2424 samples, with 269 samples designated for the testing set.

### 3.2 Implementation details

This article used the Pytorch framework to develop MD-UNet. The training and testing platform is the Ubantu18.04 system, the graphics card is GTX1070Ti, and the video memory is 10G. This article uses the Adam optimizer with a learning rate of 0.001 and a momentum of 0.9. Since the neural network is very unstable at the beginning of training, a corresponding training strategy, namely cosine annealing learning, is added to reduce the risk of over fitting, so that the model has strong robustness and good convergence to occlusion. In the cosine annealing strategy, the learning rate is reduced in the form of a cosine function, which ensures a smoother learning rate reduction and prevents the model from failing to converge due to the learning rate dropping too fast. The minimum learning rate is 0.00001. The batch size is set to 32. This paper trains MD-UNet up to 500 times in total.

### 3.3 Loss function

In medical image segmentation, the variations in the shape and size of the lesion of interest can cause the loss function to sharply drop to a local minimum during the training process. This occurrence may lead to suboptimal performance and an inability of the neural network to achieve the best segmentation. To address this concern, researchers mainly use cross-entropy (42) as the criterion to assess the proximity between the actual and predicted outputs. A lower value of cross-entropy delineates a more accurate prediction by the model. Additionally, the Dice coefficient is a standard metric to evaluate the segmentation effect and quantify the disparity between segmentation results and labels (43). Given the imbalanced nature of medical image datasets, the usage of Dice loss (DL) as a loss function is prevalent in segmenting ROI lesions and handling background imbalances. DL effectively reduces segmentation bias. The Equations of Binary Cross Entropy (BCE) and DL are as shown in Equation 7 and Equation 8.


(7) $$L_{BCE}\left(y,\hat{y}\right)=-\left(ylog\left(\hat{y}\right)+\left(1-y\right)\text{log}\left(1-\hat{y}\right)\right)$$

(8) $$DL\left(y,\hat{y}\right)=1-\frac{2y\hat{y}+1}{y+\hat{y}+1}$$

Where y represents the actual value and $\hat{y}$ represents the predicted result. In this paper, MD-UNet is trained using a combination of binary cross-entropy (BCE) and dice loss. The Equation for the loss L between the predicted value $\hat{y}$ and the target value y is shown in Equation 9.


(9) $$L=L_{BCE}\left(y,\hat{y}\right)+DL\left(y,\hat{y}\right)$$

### 3.4 Evaluation metrics

This paper compares the performance of MD-UNet with recent widely used medical image segmentation frameworks. The parameters compared in the experiment are IoU, Dice, number of parameters and computational complexity. Among them, the computational complexity is calculated according to the number of floating-point operators (FLOPs), and the Equation is shown in Equation Equation 10 and Equation 11.


(10) $$IoU=\frac{TP}{TP+FN+FP}$$

(11) $$Dice=\frac{2\times TP}{2\times TP+FN+FP}$$

Where TP , TN , FP and FN respectively represent True Positives, True Negatives, False Positives and False Negatives.

### 3.5 Experimental results

The results of this study are presented in **Table 2**. It can be seen that our proposed network exhibits significant improvement in segmentation accuracy compared to other networks, measured through Dice, IoU, parameter count, and GFLOPs (highlighted in bold in **Table 2**). Specifically, when compared to the state-of-the-art network Cenet, MD-UNet achieves a higher IoU and Dice of 4.86% and 4.84%, respectively, on the PanNuke dataset. On the ISIC2018 dataset, MD-UNet achieves a higher IoU of 4.17% and Dice of 2.31% compared to Cenet. Furthermore, in terms of computational efficiency, our proposed network demonstrates the lowest GFLOPs value of 0.241, whereas Cenet has 8.9, MedT has 21.245, and UNet has 55.840. Regarding parameter count, our proposed network has only 0.73M parameters, whereas Cenet has 29M parameters, MedT has 1.60M parameters, and UNet has 31.13M parameters. These findings indicate that MD-UNet not only outperforms other networks in terms of segmentation performance but also stands out as the most lightweight network, which refers to a network that requires fewer parameters and lower computational complexity.

**Table 2:** Performance comparison with state-of-the-art network models.

Networks	Params (in M)	GFLOPs	PanNuke		ISIC2018	
			IoU(%)	Dice(%)	IoU(%)	Dice(%)
Segnet (12)	29.40	1880.07	69.99	81.20	54.41	64.71
UNet (1)	31.13	55.84	71.99	83.60	74.55	84.03
R2UNet (14)	39.09	152.9	70.31	81.99	75.64	84.46
UNet++ (2)	9.16	34.65	72.42	83.82	75.12	84.96
UCTransNet (13)	66.24	32.98	72.43	84.19	80.73	89.82
Atttention UNet (30)	34.88	66.63	72.732	83.71	88.21	93.35
MedT (23)	1.60	21.24	73.49	83.38	88.54	93.53
UNeXt (29)	1.47	0.57	71.34	83.33	88.81	94.06
Cenet (24)	29.00	8.90	76.60	85.00	92.42	96.01
**MD-UNet**	**0.73**	**0.241**	**81.46**	**89.84**	**96.59**	**98.32**

The runtimes of different methods are presented in **Table 3**. From the table, it is evident that MD-UNet has the shortest runtime at 15.92ms (highlighted in bold in **Table 3**), while Cenet, UNeXt, and MedT exhibit runtimes of 18.71ms, 17.07ms, and 89.35ms. These findings highlight MD-UNet’s superior runtime performance, rendering it a more real-time and efficient option.

**Table 3:** Runtime comparison between different networks.

Networks	Inference Speed (in ms)
Segnet (12)	480.87
UNet (1)	27.67
R2UNet (14)	60.45
UNet++ (2)	36.59
UCTransNet (13)	350.65
Atttention UNet (30)	37.66
MedT (22)	89.35
UNeXt (29)	17.07
Cenet (24)	18.71
**MD-UNet**	**15.92**

**Figure 6:**
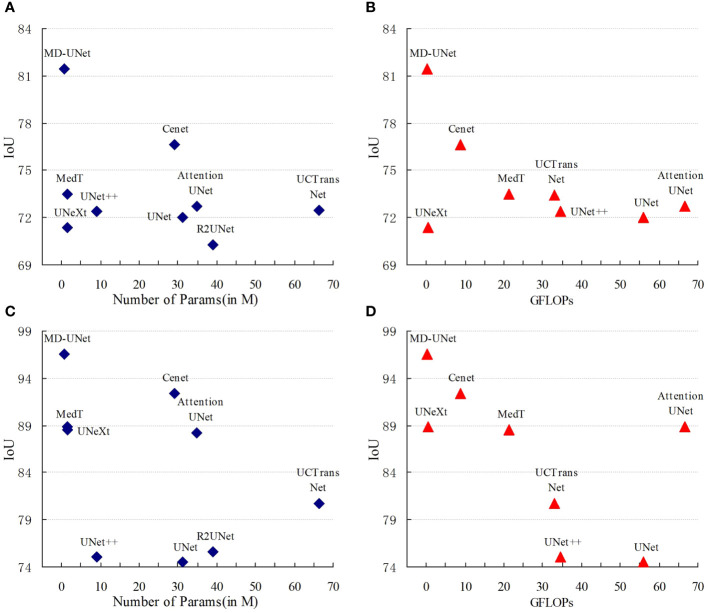
Comparison Charts. The comparison charts depict the relationship between the Dice scores (vertical axis) and the corresponding GFLOPs or parameter count (horizontal axis). Higher Dice scores indicate better performance, while lower values of GFLOPs and parameter count are preferred. The representations **(A, B)** correspond to the PanNuke dataset, whereas **(C, D)** correspond to the ISIC2018 dataset. These charts validate that MD-UNet outperforms other networks in terms of effectiveness.

**Figure 7:**
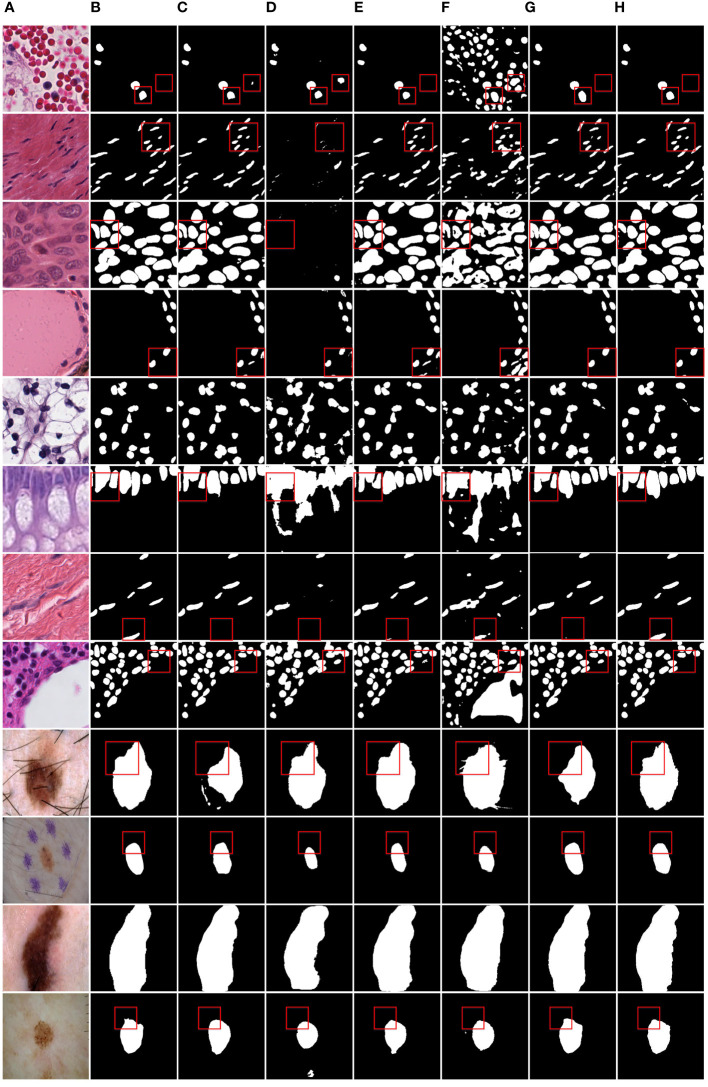
Qualitative comparison of MD-UNet on PanNuke dataset and ISIC2018 dataset. **(A)** Input, **(B)** Group Truth, **(C)** MedT, **(D)** UNeXt, **(E)** UNet, **(F)** R2UNet, **(G)** CENet, **(H)** MD-UNet.

**Figure 6** shows the IoU score plotted against the number of parameters or GLOPs. The graph reveals that MD-UNet outperforms other methods in terms of segmentation performance, computational complexity, and the number of parameters. To illustrate the improvement of our model compared to the baseline, we provide qualitative comparisons of the ISIC2018 and the PanNuke datasets in **Figure 7**. The results indicate that MD-UNet generates segmentation predictions with a competitive edge detail that is closer to the ground truth than that of other methods.

**Figure 8:**
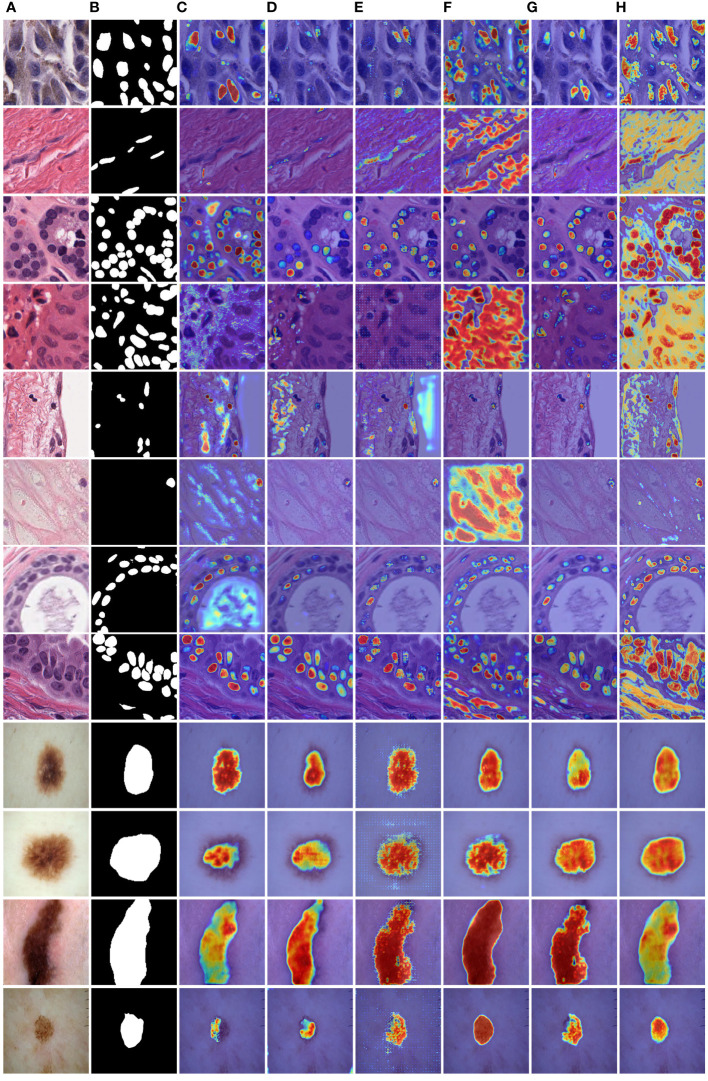
Comparison of heatmaps among various networks on the PanNuke datasets and ISIC2018 datasets. These are heatmaps of the final layers for each network. **(A)** Input, **(B)** Group Truth, **(C)** MedT, **(D)** UNeXt, **(E)** UNet, **(F)** R2UNet, **(G)** CENet, **(H)** MD-UNet.

To provide a visual comparison between MD-UNet and various state-of-the-art networks, this study includes semantic segmentation result images and heatmaps for MD-UNet and other networks, as depicted in **Figure 7** and **Figure 8**. The first eight rows correspond to the experimental comparisons on the PanNuke dataset, while the last four rows represent the comparisons on the ISIC2018 dataset. From the figures, it is evident that MD-UNet demonstrates a strong consistency between the predicted boundaries and the ground truth for different cell types and skin lesions of varying shapes. Compared to other advanced networks, MD-UNet exhibits higher accuracy and smoother segmentation results.

Specifically, for cell nuclei segmentation, the first row reveals that MD-UNet successfully eliminates interference from external objects (e.g., circular objects). Furthermore, the second, third, and fourth rows demonstrate MD-UNet’s precise segmentation of elongated, circular, and elliptical cell shapes, respectively. Even for irregularly shaped cells, as shown in the seventh row, MD-UNet still displays a certain degree of segmentation capability. Regarding skin lesion segmentation, the ninth and tenth rows illustrate MD-UNet’s ability to achieve high-quality segmentation even in the presence of hair follicle interference. Additionally, the eleventh and twelfth rows indicate MD-UNet’s ability to achieve high-quality segmentation for objects of different colors.

## 4 Ablation study

In this study, we developed a neural network based on the DA Block and MFE modules, with a U-shaped architecture consisting of five Token-MLP layers. The ablative experiments aimed to evaluate the impact of the DA Block and MFE modules on the neural network’s performance.

During the ablative experiments, our primary focus was on the DA Block and MFE modules. To assess their contributions to the neural network, we conducted individual ablative operations and observed changes in network performance. Firstly, we conducted an ablation experiment by removing the MFE module from the neural network. The results of the ablation experiment revealed that the IoU metrics of the neural network decreased by 3.44% and 1.01% on the PanNuke and ISIC2018 datasets, respectively, while the Dice metrics decreased by 2.23% and 0.91%. Subsequently, we performed a similar ablation experiment on the DA Block to gain a deeper understanding of its contribution to diagnostic performance. The experimental findings indicated that upon removing the DA Block, the neural network experienced a decrease of 3.03% and 1.00% in the IoU metrics, and a decrease of 1.93% and 0.60% in the Dice metrics, on the PanNuke and ISIC2018 datasets, respectively.

**Figure 9:**
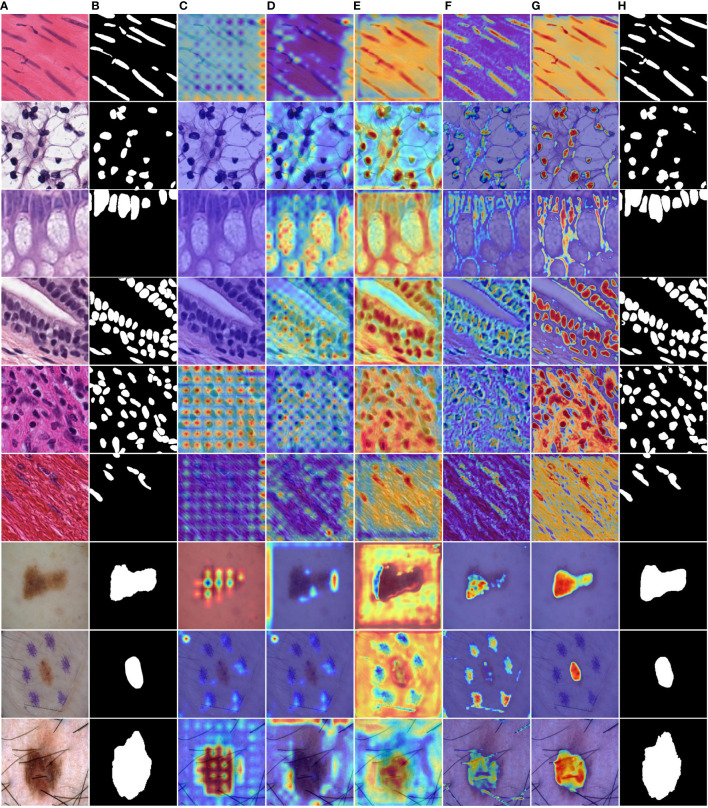
MD-UNet heatmaps at different stages on the PanNuke and ISIC2018 datasets. **(A)** Input, **(B)** Ground Truth, **(C)** Heatmap of the 5th layer of the MFE module, **(D)** Heatmap of the 5th layer of the DA Block, **(E)** Heatmap of the 1st layer of the MFE module, **(F)** Heatmap of the 1st layer of the DA Block module, **(G)** Heatmap of the final 1x1 convolution layer, **(H)** Final segmentation result of MD-UNet.

To enhance the illustration of the influence of the DA Block and MFE modules on the neural network's performance, we have visualized the experimental results, as depicted in **Table 4**. The final row corresponding to MD-UNet in **Table 4** has been bolded. Additionally, to showcase the influence of each module at different stages, we generated heatmaps of each module, as depicted in **Figure 9**. It can be observed that during the decoding stage, the MFE module captures more meaningful features, while the DA Block integrates these features with semantic features from the encoder end, eliminating ambiguity. Furthermore, columns 2, 3, 4, and 5 demonstrate that as the network output progresses, the captured semantic features become more accurate and extensive. Our experimental results clearly depict the performance differences before and after ablative experiments, as well as the disparities in heatmaps at different stages, further elucidating the roles and importance of these two modules.

**Table 4:** Quantitative analysis results of ablation experiments.

Network	Params (in M)	GFLOPs	PanNuke		ISIC2018	
			IoU (%)	Dice (%)	IoU (%)	Dice (%)
Baseline	0.23	0.116	70.82	82.95	87.56	93.46
Baseline+DA	0.64	0.136	78.02	87.61	94.57	97.42
Baseline+MFE	0.30	0.191	78.43	87.91	95.58	97.73
**Baseline+DA+MFE(Ours)**	**0.73**	**0.241**	**81.46**	**89.84**	**96.58**	**98.33**

By comprehensively analyzing the results of the ablative experiments, we conclude that the DA Block and MFE modules play a significant role in the neural network, exerting a notable influence on the overall performance of cell nuclei segmentation. These findings from the ablative experiments provide robust support for gaining a deeper understanding of the neural network’s role in diagnosing skin diseases.

## 5 Discussion

We proposed a MD-UNet network for the segmentation of tumor cell nuclei and skin lesions. The proposed method integrates several modules, including the Token-MLP block, DA block, and MFE module, within the network design. The core architecture of the proposed network mainly consists of a Multi-layer Feature Extraction (MFE) module for capturing semantic information about the shapes of the targets, and a Depthwise Attention (DA) block for effectively integrating semantic information from both the encoder and decoder outputs.

Compared to UNet, we replace the combination of convolution, batch normalization, and ReLU with the Token-MLP module, reducing the network’s parameter count and computational complexity. Furthermore, in comparison to Attention UNet, the improved DA block efficiently integrates semantic information from both the encoder and decoder, reducing semantic information loss. Additionally, to segment irregularly shaped cell nuclei and skin lesions, the introduced MFE module is employed to capture semantic information from the decoder end, allowing the network to better understand and interpret the semantic features of objects with different shapes.

While the proposed MD-UNet achieves the highest segmentation accuracy compared to state-of-the-art networks on two datasets, our approach still requires improvement. For example, in scenarios where the segmentation of cell nuclei edges and tissue boundaries in images is unclear, there is room for further refinement in the future development of our method.

## 6 Conclusion

The proposed MD-UNet in this paper is a U-shaped encoder-decoder neural network that incorporates the Token-MLP module, DA block, and MFE module. The network is built upon a backbone of five layers of Token-MLP blocks. A novel DA block is introduced to integrate semantic information from both the encoder and decoder outputs. Furthermore, a Multi-layer Feature Extraction module is devised to capture semantic information specifically from the decoder end. Asymmetric convolutions are employed instead of symmetric convolutions to enhance the ability of feature extraction for objects with varying shapes. The performance of MD-UNet is evaluated on the PanNuke dataset and the ISIC2018 dataset. Experimental results demonstrate that MD-UNet outperforms other state-of-the-art networks in terms of performance while also exhibiting fewer parameters and floating-point operations.

## Data availability statement

The original contributions presented in the study are included in the article/supplementary material. Further inquiries can be directed to the corresponding author.

## Author contributions

YC: Data curation, Methodology, Validation, Visualization, Writing – original draft, Writing – review & editing. XS: Conceptualization, Funding acquisition, Methodology, Software, Supervision, Writing – review & editing, Writing – original draft. YD: Methodology, Validation, Writing – original draft. YW: Methodology, Validation, Writing – original draft. JZ: Methodology, Validation, Writing – original draft. YZ: Supervision, Writing – review & editing.
